# Fermentation properties and functional stability of dough starter Jiaozi and Laomian after frozen storage

**DOI:** 10.3389/fmicb.2024.1379484

**Published:** 2024-04-12

**Authors:** Haifeng Li, Yulan Lv, Yingmiao Zhang, Xifeng Wang, Xiaohong Yang, Jianhang Qu

**Affiliations:** ^1^School of Biological Engineering, Henan University of Technology, Zhengzhou, China; ^2^College of Basic Medicine, Hebei Medical University, Shijiazhuang, China

**Keywords:** storage, dough, frozen, metagenomic sequencing, fermentation

## Abstract

**Purpose:**

This study aims to investigate the effects of frozen storage on the stability of traditional dough starters in China.

**Methods:**

The microbial community structure and abundance of related metabolic genes in different fermented sourdough prepared by Jiaozi (JZ) and Laomian (LM) starters before and after frozen storage at −20°C for half a year were analyzed using the shotgun metagenomic sequencing method, and differences in characteristics of texture in steamed bread were also compared by formal methods.

**Results:**

The fermentation ability (FA) and metabolic activities of yeast in the JZH sourdough (started by JZ which was stored at −20°C for half a year) were better than those of LMH sourdough (started by LM which was stored at −20°C for half a year). The dominant genera of *Acetobacter* were found to be increased in the JZH0 sourdough (started by JZH and fermented for 0 h) and those of *Lactobacillus* were found to be decreased. *Lactobacillus* (98.72%), *Pediococcus* (0.37%), *Saccharomyces* (0.27%), and *Acetobacter* (0.01%), were dominant in sourdough LMH0 (started by LMH and fermented for 0 h). The abundances of “oxidative phosphorylation-related enzymes” and the “biosynthesis of glutamate”-related enzymes and genes related to “biosynthesis of glutamate” and “unsaturated fatty acid” were higher in JZH0 than in the JZ0 sourdough (started by JZ without being frozen and fermented for 0 h). The good FA of yeast, the acid production capacity of bacteria in the sourdough, and the quality of the JZH steamed bread (made by the JZH starter) indicated the better freezing tolerance of the microorganisms in JZ than in LM.

**Conclusion:**

The conclusion of this study suggests the better application potential of the JZ as the fermentation starter in actual production.

## Introduction

Chinese steamed bread (CSB) is a popular food made from wheat flour fermented by traditional starters or yeast (Li et al., 2017). Sourdough fermentation may improve the bioavailability of the dietary fiber complex and the uptake of minerals (Gobbetti et al., 2014) and types of microorganisms that were involved with different metabolic pathways and substances produced during the fermentation process, thereby resulting in significant differences in terms of taste, texture characteristics, and flavor of steamed bread (Yan et al., 2019; Liu et al., 2020b). Hence, the type of dough fermentation starters used is one of the key factors affecting the quality of the fermented dough and steamed bread.

In addition, Jiaozi (JZ) and Laomian (LM) are two of the main traditional fermentation starters applied in the manufacturing process of CSB. JZ is usually prepared by raw materials composed of Chinese *DaQu*, maize flour, and muskmelon and the dry powder of JZ, rice wine, Chinese *DaQu*, and rice flour. Moreover, the initial pH of JZ usually is >5. However, in general, the LM refers to the part of the sourdough with good acidification and leavening capacity that remained for the next process of dough fermentation, with wheat used as the raw material and an initial pH of about 4. The functional microorganisms in JZ starters mainly include yeasts, acetic acid bacteria (AAB), and lactic acid bacteria (LAB), while yeasts and LAB are dominant functional microorganisms in LM (Li et al., 2017).

Moreover, the stability and properties of dough starters are mainly affected by the stability of the microorganisms, different compositions of raw materials, and the manufacturing process (Minervini et al., 2014; Liu et al., 2018). Interactions between different microorganisms and the enzymatic metabolism inherent in grain flour can give rise to volatile compounds like acids, alcohols, and esters in steamed bread (Coda et al., 2014). Therefore, microbial diversity and its stability are extremely important for enhancing fermented products.

In daily life, fermentation starters often cannot be used all at once and need to be stored under frozen conditions because the subzero storage temperatures could prolong microbial shelf life and retard staling in the dough (Bosmans et al., 2014). Freeze-drying is a realistic technology for the stability and maintenance of the potential sourdough starters *Lactobacillus fermentum* and *Wickerhamomyces anomalus* for a long time; however, the choice of the cryoprotectant will influence the effectiveness of the process (Stefanello et al., 2019).

Similar to that of the frozen dough, the activity, vitality, and quantity of the yeasts (Luo et al., 2018), LAB, as well as AAB in the JZ and LM starters are possibly inhibited and reduced during frozen storage. In our previous study, the fermentation ability (FA) of yeast in LM almost decreased to 0, while an FA of more than 40% was maintained in JZ after storage at −20°C for 180 days. Thus, the stability and properties of the dough starter and the texture, color, and flavor of the CSB will be influenced to some extent; hence, freezing tolerance is necessary for the microorganisms in starters under frozen storage conditions.

In the previous study, the research on traditional starters for sourdough focused mainly on the microbial species in fermented dough and the production of steamed bread. There are few reports on the storage methods for various traditional starters and the effects of frozen storage conditions on the stability of different starters. In this study, the sourdoughs were fermented with two traditional starters (JZ and LM), which were stored for 0 and 180 days at −20°C, respectively. Furthermore, the diversity of microbial taxa and their functional stability and gene abundances in the sourdough samples fermented for 0 and 8 h were investigated using the plate counting assay and shotgun metagenomic sequencing method.

## Materials and methods

### Materials

The JZ sample was obtained from Shangqiu City, Henan, China, and the LM sample was obtained from Dezhou City, Shandong, China. Both samples were placed in sealed plastic bags on ice and transported immediately to the laboratory. Then, a part of the JZ and LM samples was stored at −20°C in dark conditions and used as starters in JZH0, JZH8, LMH0, and LMH8 treatments, and the other part of each fresh sample was used as fermentation starters in JZ0, JZ8, LM0, and LM8 treatments. In addition, the commercial wheat flour (0.38% ash, 10.89% protein, and 13.79% moisture) used in this study was obtained from Jinyuan Flour Co., Ltd. (Zhengzhou, China).

### Sourdough fermentation

Based on the recommendation of each manufacturer of JZ and LM, 50 mL of aseptic water and 100 g of wheat flour were mixed with 10 g of the JZ and LM starters, respectively. The doughs prepared with the JZ and LM starters were fermented at 30°C and 85% relative humidity for 8 h. JZ0 (or LM0) and JZ8 (or LM8) were referred to as the sourdoughs prepared using JZ (or LM) as the dough starter and fermented for 0 or 8 h.

After 180 days of storage at −20°C, 20–100 g of the frozen starters (named JZH or LMH) was taken and thawed at 37°C for about 30 min. In addition, JZH0 (or LMH0) and JZH8 (or LMH8) samples were the sourdoughs prepared using JZH (or LMH) as starters and fermented for 0 or 8 h. The dough preparation and fermentation conditions were maintained the same as that of sourdough JZ0 (or LM0) and JZ8 (or LM8).

### Chemical analyses

The pH and total titratable acidity (TTA) of the samples used in this study were determined with the method previously described (Li et al., 2014). The 10-g samples were homogenized with 90 mL of ddH_2_O. Subsequently, the pH value was recorded, and the TTA was expressed in milliliters of 0.1 N NaOH, and a pH of 8.5 was obtained. Therefore, to evaluate the fermentation ability (FA) of the sourdough, 100 g of the dough was stuffed into a 500-mL measuring cylinder immediately after being prepared with JZ, LM, or JZH and LMH starters, and the volume from the starting point (100 mL) was recorded at a proper interval to determine the increase in volume of the sourdough, which was used to evaluate the FA of each starter (Suo et al., 2020).

### Microbiological analysis and counting

A total of 1 g of the samples was taken and suspended in 9 mL of sterile 0.85% NaCl solution and shaken for 60 min in an air shaker at 0 or 8 h for fermentation. Then, the suspension was serially diluted 10-fold in the sterile 0.85% NaCl saline solution. Then, 200 μL of suitable dilutions were plated on yeast peptone dextrose (YPD) or De Man–Rogosa–Sharp (MRS) agar and incubated under 28 or 37°C for yeast and LAB number counting, respectively.

### DNA extraction and shotgun metagenomic sequencing

The genomic DNA for metagenomics sequencing analysis was extracted from the different eight sourdough samples (mixtures of three replicates from each treatment) by following the instructions of the Universal Genomic DNA Extraction Kit (Solarbio Science & Technology Co., Ltd., Beijing, China). In addition, the concentration and purity of the extracted DNA were assessed with 1% agarose gel electrophoresis using a NanoDrop 2000 spectrophotometer (Thermo Fisher Scientific, USA). DNA was fragmented to an average size of about 400 bp using Covaris M220 (Gene Company Limited, China) for paired-end library construction. Paired-end libraries were first prepared by using the TruSeq^TM^ DNA Sample Prep Kit (Illumina, San Diego, CA, USA). Then, the adapters containing the full complement of sequencing primer hybridization sites were ligated to the blunt-end fragments. Paired-end sequencing was performed on the Illumina HiSeq4000 platform (Illumina Inc., San Diego, CA, USA) at Majorbio Bio-Pharm Technology Co., Ltd. (Shanghai, China) using the HiSeq3000/4000 PE Cluster Kit and HiSeq3000/4000 SBS Kit according to the manufacturer's instructions (www.illumina.com). Sequence quality control and genome assembly were conducted by SeqPrep (https://github.com/jstjohn/SeqPrep) (Li et al., 2021a; Yi et al., 2023).

Taxonomic assignment was generated by BLASTP (Version2.2.28+, http://blast.ncbi.nlm.nih.gov/Blast.cgi) against the NCBI-NR database, with a cut-off E-value of 1e^−5^, and the taxon abundance of each sample was distributed into the family, genera, and species levels. Open-reading frames (ORFs) of all the contigs (>100 bp) were predicted, retrieved, and translated to amino acid sequences using MetaGene (http://metagene.cb.k.u-tokyo.ac.jp/) for functional annotation. Furthermore, for microbial community function analysis, all ORFs were annotated using BLAST (cut-off E-value of 1e^−5^) against the Kyoto Encyclopedia of Genes and Genomes (KEGG) databases by BLASTP (Version 2.2.28+, http://blast.ncbi.nlm.nih.gov/Blast.cgi). The annotated results including KEGG orthology (KO), pathways, and module information were also obtained for further analysis. All the metagenomic sequencing raw data have been deposited in the NCBI Sequence Read Archive database (https://www.ncbi.nlm.nih.gov/sra), and the accession numbers of the eight sourdough samples, namely, LM0, LM8, LMH0, LMH8, JZ0, JZ8, JZH0, and JZH8 were SRR23389164 (PRJNA931763), SRR23389231 (PRJNA931763), SRR23418126 (PRJNA931780), SRR23418159 (PRJNA931782), SRR23418172 (PRJNA931449), SRR23606465 (PRJNA931577), SRR23389426 (PRJNA931774), and SRR23606464 (PRJNA931774), respectively.

A total of 372, 111, and 330 clean reads were generated from the eight samples. In addition the differences in microbiome composition between the sourdoughs prepared by starters before (JZ0 and LM0) and after (JZH0 and LMH0) frozen storage were analyzed by Fisher's exact test (at 95% confidence intervals).

### The preparation of steamed bread

Steamed bread was prepared according to the slightly modified method used by Li et al. (2021b). Ten grams of JZ and LM was weighed, and 50 mL water was added; then, they were activated in a fermentation cabinet (HWS180, Bilon Instrument Co., Ltd., China) at 35°C and 85% humidity for 30 min. After the activation, 100 g flour was added, a smooth dough was made, and then it was allowed to prove under the above conditions for 2 h. Then, the dough was sheeted 10 times and split into 100-g portions. The chunks were shaped into rounded forms by hand, fermented at 35°C and 85% relative humidity for 40 min, and then steamed in a pot (JYC-21HEC0, Joyong, China) for 25 min. After cooling at room temperature for 5 min, the steamed breads were taken to the subsequent analysis.

### Determination of the steamed bread texture

The specific volume of steamed bread was measured by referring to the method used by Li et al. (2021b). After the steamed bread is cooled for 1 h, its mass M (g), height H (mm) and diameter D (mm) were calculated. The volume V (mL) of the steamed bread was measured by the seed displacement method, the specific volume of steamed bread was expressed as V/M and the ratio of height to diameter was expressed as H/D. The color of steamed bread was evaluated by using the CR-400 color difference meter, and different samples were measured three times in parallel.

The texture properties of the steamed bread were analyzed by using a TA-XT2i Texture Analyser equipped with a P/36R probe (Zhu et al., 2016). The steamed bread to be measured was cut into 15-mm-thick sheets, and the hardness, resilience, elasticity, and chewability of the steamed bread were tested and analyzed by the texture analyzer mentioned previously. The speed before, during, and after the test was 3, 1, and 1 mm/s, respectively; the compression degree was 50%; and the trigger force was 5 g.

### Data analysis

All experimental data were expressed as mean ± standard deviations after three independent repetitions and statistically analyzed using the SPSS package (version 19.0 for Windows, IBM Inc., Chicago, IL, USA). The differences between the mean values were analyzed using one-way analysis of variance (ANOVA) and Duncan's test. *P* < 0.05 was considered statistically significant.

## Results

### Chemical and microbiological properties of sourdough samples

Changes in pH, TTA, and FA of the sourdough, prepared using different starters after fermentation for 0 and 8 h are summarized in Table 1. There was no significant difference in TTA between JZ8 and JZH8 or between LM8 and LMH8. However, the pH decreased by 2.3, and ~11.9 mL of the acid was produced after 8 h of fermentation for the sourdough prepared by JZ, and the variation in acid level and pH was 10.55 mL and 1.9, respectively, for that of JZH, 6.6 mL and 1.3 for that of LM, and 6.05 mL and 1.4 for that of LMH. Moreover, the pH values of sourdoughs prepared by LM and LMH starters were higher than those of the JZ and JZH, respectively, and their TTA was significantly lower than that of the JZ samples (*P* < 0.05). Moreover, there is no significant difference found in the acid production capacity between the sourdoughs prepared by JZH and JZ (or LMH and LM), but it is better for JZ than for LM.

**Table 1:** Chemical properties and quantities of yeast and LAB in the sourdough samples prepared by JZ, JZH, LM, and LMH fermented for 0 or 8 h.

**Sourdough samples**	**pH**	**TTA/mL**	**Fermentation ability (mL)**	**Yeast log cfu/g dough**	**LAB log cfu/g dough**
JZ0	6.02 ± 0.06^a^	1.70 ± 0.14^d^	100 ± 0.05^d^	7.64 ± 0.06^bc^	7.85 ± 0.04^d^
JZ8	3.72 ± 0.07^f^	13.60 ± 0.14^a^	257 ± 1.41^a^	8.13 ± 0.07^a^	9.38 ± 0.01^a^
JZH0	5.71 ± 0.05^b^	2.30 ± 0.14^d^	100 ± 0.12^d^	7.39 ± 0.12^c^	7.94 ± 0.09^cd^
JZH8	3.89 ± 0.01^ef^	12.85 ± 0.07^a^	251 ± 1.63^a^	7.93 ± 0.04^ab^	9.06 ± 0.06^b^
LM0	5.31 ± 0.05^c^	3.15 ± 0.21^c^	100 ± 0.18^d^	7.00 ± 0.00^de^	8.13 ± 0.07^c^
LM8	4.00 ± 0.07^de^	9.75 ± 0.21^b^	222 ± 4.95^b^	7.76 ± 0.12^abc^	8.95 ± 0.09^b^
LMH0	5.51 ± 0.04^b^	2.25 ± 0.07^d^	100 ± 0.23^d^	6.85 ± 0.21^e^	7.98 ± 0.03^cd^
LMH8	4.13 ± 0.01^d^	9.10 ± 0.42^b^	210 ± 0.71^c^	7.39 ± 0.12^cd^	8.83 ± 0.07^b^

The data are expressed as the mean ± standard deviation, and different letters in the same column indicate the significance of the difference (*P* < 0.05).

Furthermore, there was no significant difference found in FA between the JZ8 and JZH8 sourdough, both higher than 250 mL, thereby indicating the gas production capacity of yeast was still good after 6 months of storage at −20°C. In addition, the FA of the LMH8 sourdough was significantly lower than that of the LM8 sourdough, and the FA of JZ before and after frozen storage was significantly higher than that of LM.

#### Microbial taxonomic analyses

The number of colony-forming units (CFU) of yeast and LAB in sourdough samples was determined by the plate counting method, and the total numbers of colony-forming units of LAB and yeast in different sourdoughs are shown in Table 1. After fermentation for 8 h, the number of LAB and yeast CFUs in JZH0 and JZH8 sourdough increased from log 7.94 to 9.06 cfu/g and from 7.39 to 7.93 cfu/g, respectively, and in LMH0 and LMH8 sourdough from log 7.98 to 8.83 cfu/g and from 6.85 to 7.39 cfu/g, respectively. In addition, the number of CFUs of LAB in JZ0 sourdough was lower than that of the LM0 sourdough; however, it increased rapidly in JZ8 or JZH8 sourdough after 8 h of fermentation than in LM8 and LMH8 and produced more TTA. Thus, after frozen storage, the activity of yeast was more affected than that of LAB, particularly in the LM sourdough samples in this study.

**Figure 1:**
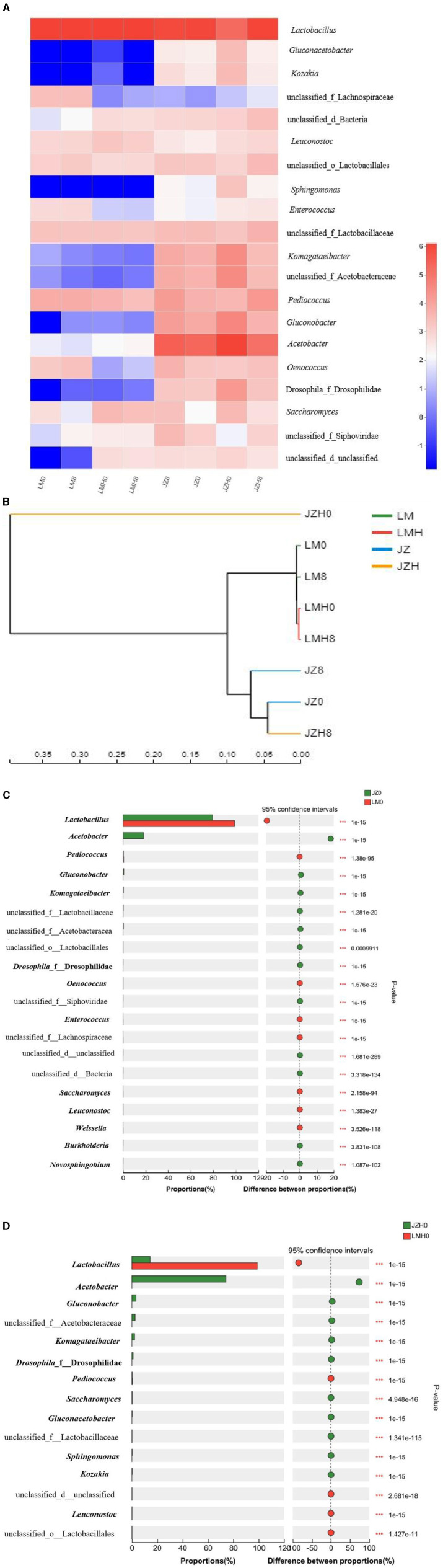
Heatmap analysis of relative gene abundance (%) at the genus (**A**) level of microbial compositions, cluster tree analysis of multiple samples (**B**), and microbiome composition of the different sourdough samples prepared by JZ and LM before (**C**) and after (**D**) frozen storage (0 and 8, fermented for 0 or 8 h; H, after frozen storage for 6 months under -20°C conditions).

In addition, the relative abundance (%) of microbial compositions at the genus level of different sourdough samples was obtained based on the results of the shotgun metagenomic sequencing method. As shown in Figure 1A, the genera such as *Acetobacter* (77.09%), *Lactobacillus* (11.68%), *Gluconobacter* (2.86%), *Komagataeibacter* (2.09), and *Saccharomyces* (0.23%) were the main microbial communities in JZH0 sourdough, and *Lactobacillus* (79.53%), *Acetobacter* (17.49%), *Pediococcus* (0.39%), and *Saccharomyces* (0.01%) were dominant in JZ0 sourdough. Moreover, *Lactobacillus* (98.72%), *Pediococcus* (0.37%), *Saccharomyces* (0.27%), and *Acetobacter* (0.01%) were dominant in LMH0 sourdough and *Lactobacillus* (98.56%), *Pediococcus* (0.54%), *Saccharomyces* (0.05%), and *Acetobacter* (0.01%) in LM0 sourdough.

*Lactobacillus* (70.26%), *Acetobacter* (24.26%), *Pediococcus* (1.18%), and *Gluconobacte*r (1.12%) were dominant in microbial communities in the JZ8 sourdough samples, and the genera such as *Lactobacillus* (85.67%), *Pediococcus* (1.4%), and *Acetobacter* (10.29%) were also dominant in the JZH8 samples. However, the *Lactobacillus* (98.46%) and *Pediococcus* (0.53%) were predominant in the LM8 sourdough. *Lactobacillus* (99.05%) and *Saccharomyces* (0.21%) were predominant in the LMH8 sourdough.

As shown in Figure 1B, the microbial composition of JZH0 is different compared to that of the JZ0; however, the strains in JZH8, JZ0, and JZ8 were gathered together, thereby indicating that the vitality of the dominant functional strains in the starter JZH recovered gradually after fermentation under appropriate conditions. However, after the same storage time and conditions, the strains in LMH0 and LMH8 have always been gathered together, which is quite different from the microbial composition in LM0 and LM8.

The differences in microbiome composition between sourdoughs prepared by JZ and LM before (JZ0 and LM0) and after (JZH0 and LMH0) frozen storage are shown in Figures 1C, D. The abundance of the *Acetobacter* genus in the JZH0 sourdough sample was higher than that in the JZ0 sourdough after frozen storage, thus indicating the better freezing resistance of AAB in the JZ starter. With the fermentation time extended to 8 h, the pH of the sourdough decreased, the proportion of LAB increased, and the content of AAB decreased in the JZH8 sample. The abundances of *Saccharomyces* was more in the LM0 sourdough than in the JZ0 sourdough, while it was greater in the JZH0 sourdough than in the LMH0 sourdough after frozen storage.

### Abundances of enzyme gene and metabolic pathways analyzed by shotgun metagenomic sequencing method

**Figure 2:**
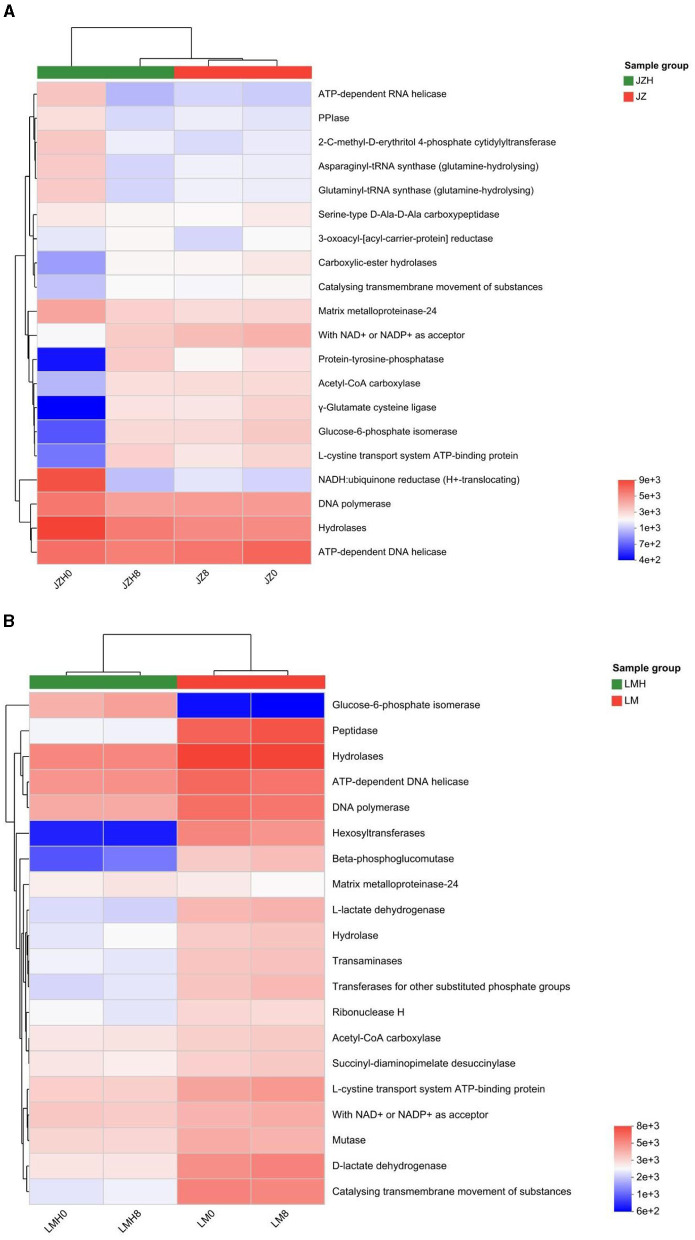
Differences of microbial enzyme gene abundances of the sourdough samples prepared by JZ **(A)** and LM **(B)** before and after frozen storage.

The microbial enzyme gene abundances are higher in JZH0 than in JZ0, including enzymes such as NADH-quinone oxidoreductase subunit A [EC 7.1.1.2], quinol oxidase cytochrome bd ubiquinol oxidase [EC 7.1.1.7], xanthine dehydrogenase [EC 1.17.1.4], xanthine dehydrogenase/oxidase, XDH/XO [EC 6.3.5.7], and they are important enzymes in prokaryotes due to their involvement in oxidative phosphorylation for energy production, as shown in Figure 2A.

In addition, the gene abundance proportions of asparaginyl-tRNA synthase (glutamine-hydrolyzing) [EC 6.3.5.6] (catalyzes reactions to synthesize L-glutamine), glutamate synthase (NADPH) [EC 1.4.1.13] (catalyzes reactions to synthesize L-glutamine), succinate-semialdehyde dehydrogenase (NADP+) [EC 1.2.1.79] (catalyzes L-glutamate to synthesize succinate), succinate-semialdehyde dehydrogenase [NAD(P)+] [EC 1.2.1.16] (catalyzes L-glutamate to synthesize succinate), and glutarate-semialdehyde dehydrogenase [EC 1.2.1.20] (an enzyme that catalyzes reactions to synthesize glutarate and NADH; https://www.genome.jp), were all larger than those in the JZ0 sourdough. When compared to that of the LM0 sourdoughs, the higher microbial enzyme gene abundances in LMH0 sourdoughs were observed in ribonucleoside-diphosphate reductase [EC 1.17.4.1], and with NAD+ or NADP+ as an acceptor [EC 1.1.1.-], as shown in Figure 2B.

**Figure 3:**
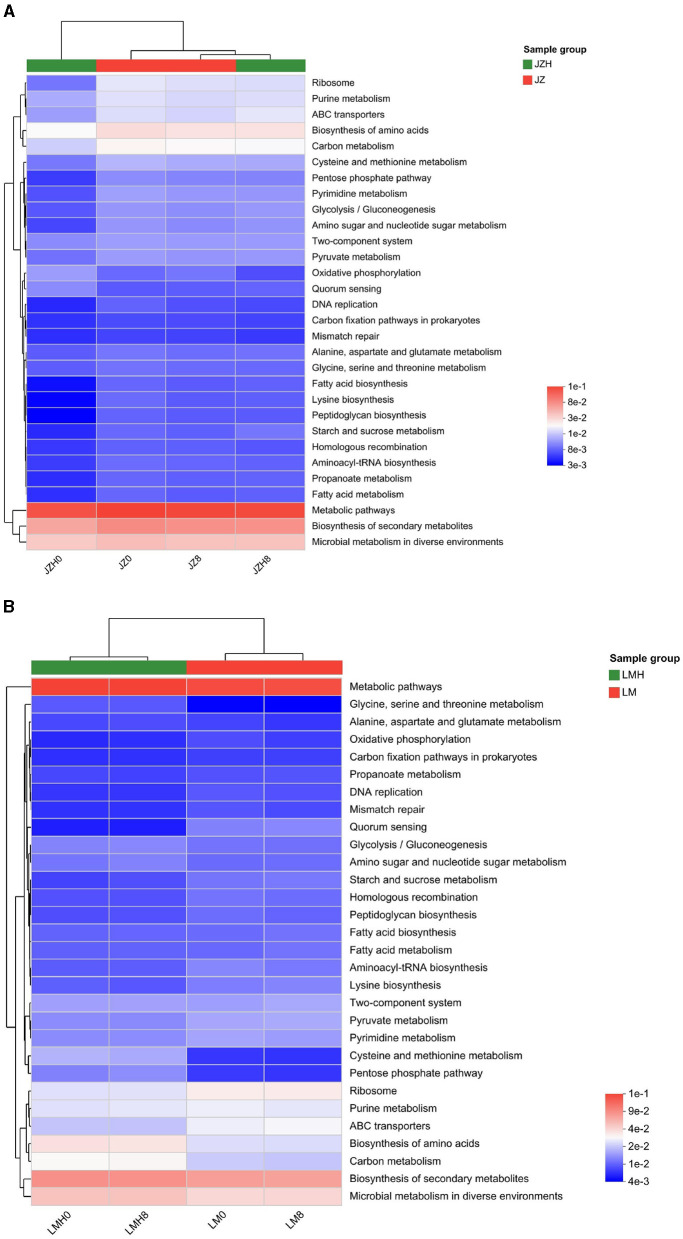
Heatmap analysis of gene abundances of “metabolic categories” in the pathway level 3 of the sourdough samples prepared by JZ **(A)** and LM **(B)** before and after frozen storage (0 and 8, fermented for 0 or 8 h; H, after frozen storage for 6 months under −20°C conditions).

In this study, the abundance of “oxidative phosphorylation” pathway genes in JZH0 (0.016%) was higher than that in the JZ0 (0.010%), while the “ABC transporter,” “ribosome,” and “carbon metabolism” gene abundances decreased in JZH0 (0.016, 0.012, and 0.023%, respectively) compared to that of the JZ0 (0.021, 0.023, and 0.029%, respectively). It also increased to the proportion as that in JZ0 after fermentation for 8 h in JZH8 (0.025, 0.023, and 0.029%, respectively; as shown in Figure 3A). Moreover, (as shown in Figure 3B), the higher gene abundances in the “biosynthesis of amino acid” and “carbon metabolism” pathways both increased in LMH0 (0.039% and 0.032%) and LMH8 (0.040% and 0.033%) compared to that in the LM0 (0.027% and 0.023%), which did not undergo freezing storage. In addition, the proportions of “ribosome,” “ABC transporter,” and “pyruvate metabolism” decreased in the LMH0 sourdough (0.026, 0.021, and 0.014%, respectively) compared to the LM0 sourdough (0.039, 0.030, and 0.018%, respectively), whereas it did not increase in the LMH8 sourdough (0.026, 0.021, and 0.014%, respectively) after fermentation for 8 h, indicating the microbial activity could not return to the level observed before freezing.

**Figure 4:**
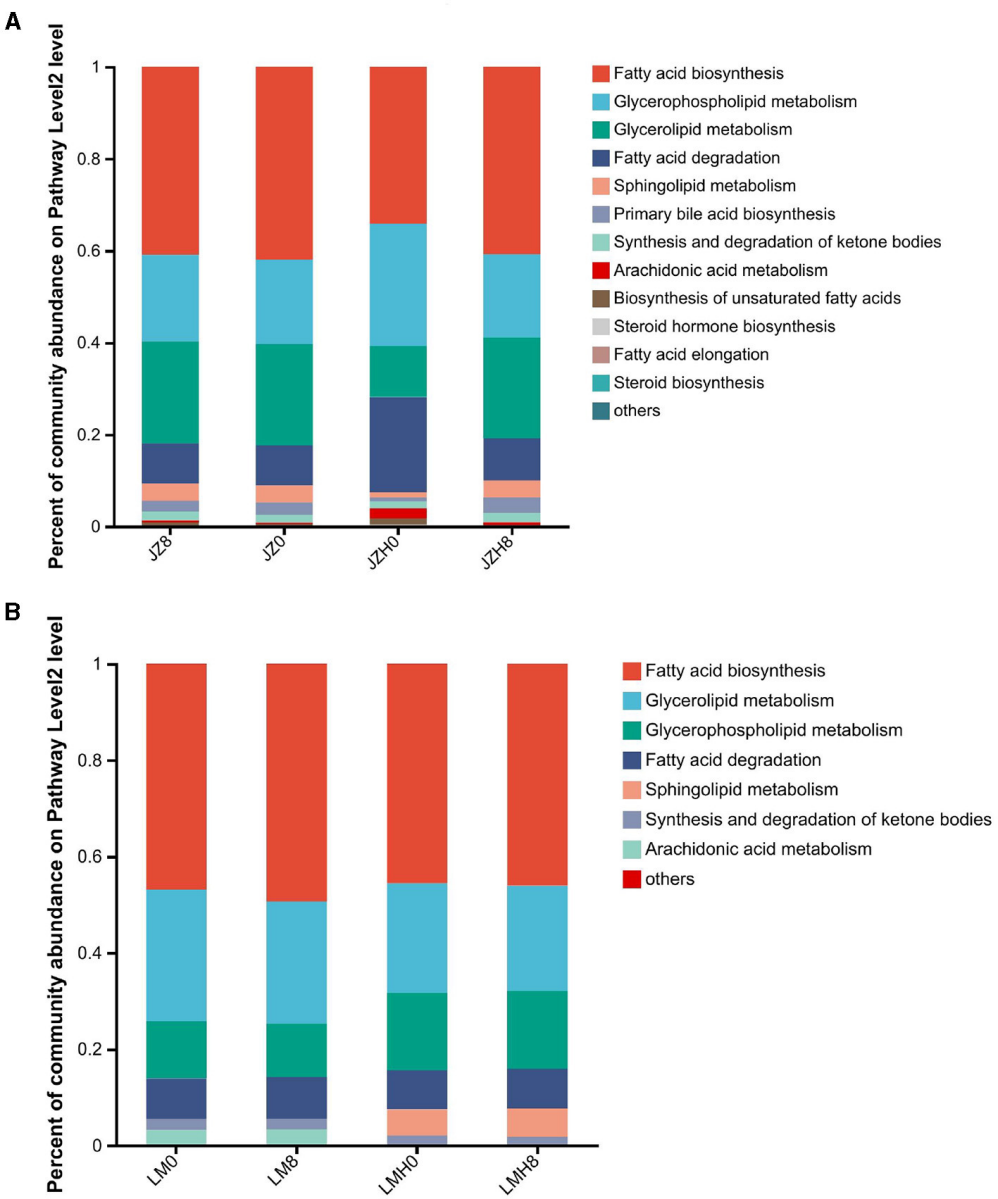
Differences in gene abundances of “fatty acid metabolism” in the pathway level 2 of the sourdough samples started by JZ **(A)** and LM **(B)** before and after frozen storage (0 and 8, fermented for 0 or 8 h; H, after frozen storage for 6 months under −20°C conditions).

For the lipid metabolism (KEGG level 2) gene abundance proportions between the samples before and after frozen storage (as shown in Figure 4A), the proportions of glycerophospholipid metabolism (ko00564)-, fatty acid degradation (ko00071)-, arachidonic acid metabolism (ko00590)-, and biosynthesis of unsaturated fatty acids (ko01040)-related gene abundances were higher in JZH0 sourdough than in the JZ0 sourdough. Moreover, the proportions of glycerophospholipid metabolism (ko00564)- and sphingolipid metabolism (ko00600)-related gene abundances in LMH0 were higher than those in the LM0 (Figure 4B). In this study, the proportions of “biosynthesis of unsaturated fatty acid” (ko01040)-related gene abundances increased particularly in the JZH0 sourdough samples as compared to that of the JZ0 sourdough, while it was not evident in the LMH sourdough (Figure 4B).

**Figure 5:**
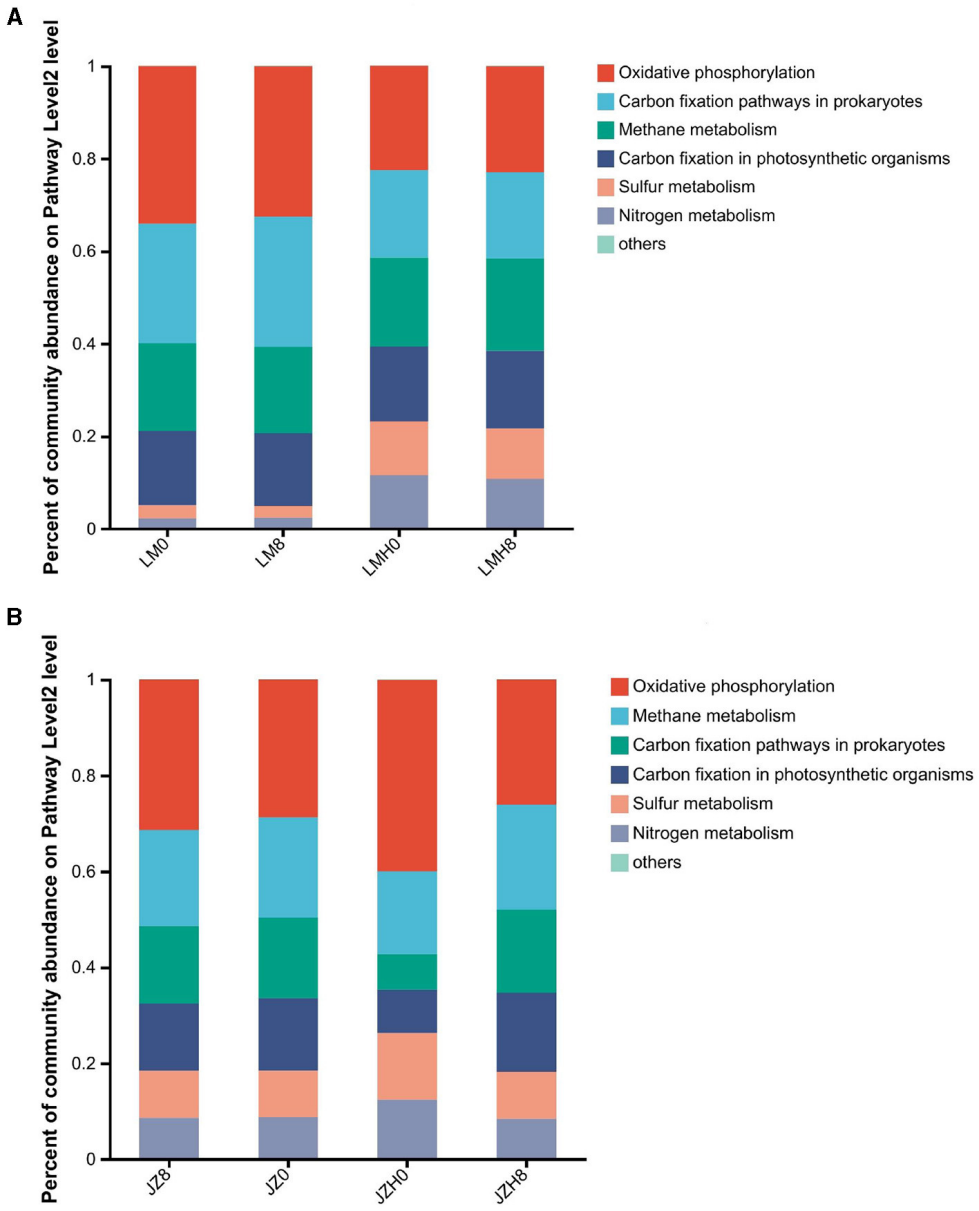
Differences of gene abundances of “energy metabolism” in the pathway level 2 of the sourdough samples started by JZ **(A)** and LM **(B)** before and after frozen storage.

For the differences in “energy metabolism” gene abundance proportions between the sourdough samples before and after frozen storage, the gene abundance proportions for oxidative phosphorylation (ko00190), nitrogen metabolism (ko00910), and sulfur metabolism (ko00920) were obviously larger in the JZH0 sourdough than in the JZ0, as shown in Figure 5A. Moreover, the gene abundance proportions of energy metabolism sulfur metabolism (ko00920) and nitrogen metabolism (ko00910) were larger in the LMH0 sourdough than in the LM0 sourdough (Figure 5B).

### Quality of the different kinds of steamed bread

As shown in Table 2, the specific volume of steamed bread prepared by JZ is the largest, 2.38, whereas that of the LMH is the smallest, 1.66. The height–diameter ratio of the LM steamed bread (0.78) was larger than that of the JZ steamed bread (0.61); however, there was no significant difference found between the steamed bread started with the same kind of starter before and after frozen storage. In addition, the hardness of the LMH steamed bread is the highest, 4,157, whereas that of JZ steamed bread is the lowest, 2,175 (Table 3).

**Table 2:** The influence of frozen storage on the specific volume, ratio of height to diameter, and color of the steamed breads prepared from JZ, JZH, LM, and LMH sourdoughs.

**Starters**	**Specific volume**	**Ratio of height to diameter**	*L* ^*^	a^*^	b^*^
JZ	2.38 ± 0.11^a^	0.61 ± 0.02^b^	81.70 ± 0.06^b^	2.59 ± 0.01^b^	12.10 ± 0.26^a^
JZH	2.13 ± 0.04^ab^	0.62 ± 0.00^b^	80.82 ± 0.94^b^	2.75 ± 0.02^ab^	13.05 ± 0.11^a^
LM	1.90 ± 0.05^bc^	0.78 ± 0.02^a^	87.80 ± 0.13^a^	3.00 ± 0.23^ab^	11.12 ± 2.57^a^
LMH	1.66 ± 0.10^d^	0.75 ± 0.01^a^	86.54 ± 0.42^a^	3.24 ± 0.20^a^	10.02 ± 2.04^a^

The data are expressed as the mean ± standard deviation, and different letters in the same column indicate the significance of the difference (*P* < 0.05).

**Table 3:** The influence of frozen storage on the texture characteristics of the steamed breads prepared from JZ, JZH, LM, and LMH sourdoughs.

**Textural properties**	**Hardness (g)**	**Resilience (%)**	**Springiness (%)**	**Chewiness (g)**
JZ	2,175.21 ± 32.54^d^	47.51 ± 0.39^a^	94.07 ± 0.91^a^	1,614.58 ± 30.86^d^
JZH	2,572.31 ± 6.71^c^	45.15 ± 0.26^b^	92.90 ± 0.41^a^	1,854.99 ± 26.17^c^
LM	3,498.94 ± 17.87^b^	44.68 ± 0.16^b^	93.24 ± 0.84^a^	2,488.49 ± 56.29^b^
LMH	4,157.92 ± 36.55^a^	41.15 ± 0.98^c^	91.51 ± 0.46^a^	2,829.11 ± 54.36^a^

The data are expressed as the mean ± standard deviation, and different letters in the same column indicate the significance of the difference (*P* < 0.05).

As shown in Supplementary Figure 1, the appearance of the JZH steamed bread was better than that of the LMH steamed bread, among which the front and side surface and the longitudinal section of the JZH steamed bread were smoother and uniform. However, the volume and texture properties of the LMH steamed bread were worse as compared to that of the other samples.

## Discussion

The initial pH of the sourdough prepared by LM is usually lower than that of JZ, and the sourdough prepared by JZ had a lower TTA than that of the LM (Li et al., 2017). However, the pH of the sourdoughs of JZ8 and JZH8 was lower than that of the LM8 and LMH8 sourdough, indicating the better acid production capacity of the microorganisms maintained in the JZH starter after frozen storage. The increase in LAB and yeast cells is consistent with the results of Wang et al. (2020), and after 18 h of fermentation at 32°C, the number of viable cell counts ranged from log 8.69 to 8.95 cfu/g and from 7.70 to 8.00 cfu/g for LAB and yeasts (after the freeze-drying process), respectively (Cossignani et al., 1996).

Smaller abundances of yeast (*Saccharomyces* and other genera) were detected in all the samples, as compared to the results of quantitative analysis by the plate counting method in the present study. It may be due to some degradation or insufficient extraction in the process of fungal DNA extraction. Furthermore, the decrease in the survival ratio after storage in frozen conditions usually depended on the yeast strain, and the leavening ability decreased after 4 days of frozen storage (Almeida and Pais, 1996). Moreover, the sourdoughs that reactivated after 90 days of storage at −20°C hardly showed measurable leavening capacity. Freezing and thawing may cause severe injury to yeast cells and then lower the fermentation ability (Suo et al., 2018). In addition, after 28 days of frozen storage at −20°C, concentrated cells (3.2 × 10^9^ cfu/mL) grew in MRS broth cultures and showed an 84% reduction in inviable cells (Wright and Klaenhammer, 1981). The yeast (normally referred to as *Saccharomyces cerevisiae)* is always well-grown between 20 and 30°C, and its viability would be greatly inhibited below 0°C (Ji et al., 2016); hence, the freezing tolerance of most yeast species is very low. Moreover, freezing yeast in the dough is probably more damaging than freezing yeast directly because the yeast in the dough system would suffer from stress factors such as high osmotic pressure and oxidative stress (Tsolmonbaatar et al., 2016; Luo et al., 2018). The cultivation conditions also affected the quality of the dough starter cultures (Johanson et al., 2020).

An increased amount of LAB was present in sourdoughs because the bacteria such as *Lactobacillus* could adapt to more acidic conditions (Jiang et al., 2020). Therefore, highly acidic conditions could favor the growth of bacteria such as *Lactobacillus* over *Pediococcus, Leuconostoc*, and even *Acetobacter*. As the sourdough maturing progressed, dominant strains of sourdough gradually constituted the major microbiota and the microbial biodiversity decreased. All these suggested the intrinsic robustness of the microorganisms in sourdough, which contributed to resisting the invasion of foreign microorganisms (Wang et al., 2020). All the samples, except the JZH0 (genus *Acetobacter* is predominant) in this study, are characterized by a high relative abundance of LAB, which is mainly composed of the genera *Lactobacillus, Leuconostoc, Pediococcus*, and *Oenococcus*. Other genera such as *Enterobacter, Citrobacter*, and *Staphylococcus*, which may exist in various sourdough samples (Liu et al., 2016a,b), were not detected in this study, while *Enterococcus* was found. Furthermore, the genera such as *Gluconobacte*r, *Komagataeibacter*, and *Gluconacetobacter*, as well as *Kozakia* (only in sourdoughs of JZH0), were detected in the JZ-related samples, thereby indicating the special composition of AAB species (Qiu et al., 2021) for JZ. These results were in line with those of the previous studies by Li et al. (2021a). Moreover, in other JZ samples, *Lactobacillus, Weissella, Acetobacter*, and *Sphingomonas* were identified as the predominant bacterial genera, and *Acetobacter* was dominant in the samples of KEL (from Xinjiang Province) and YA (from Shanxi Province; Liu et al., 2020b). The tolerance of microorganisms to freezing-induced injury might be related to the characteristics of different strains (Liu et al., 2020a) and perhaps the microbial composition and materials in the starters. Maize flour is a promising and low-cost carrier material for producing freeze-dried starter cultures (Houngbédji et al., 2020), and the JZ samples selected in this study were prepared from maize flour.

Synthesis of L-proline from L-glutamate could be catalyzed by γ-glutamyl kinase and γ-glutamyl phosphate reductase. Yeast cells could biosynthesize proline from glutamate in the cytoplasm for freeze tolerance in frozen doughs, and the same pathway was also found in bacteria and plants (Watanabe et al., 2018). When genes involved in the oxidative phosphorylation of a yeast strain are relatively strongly expressed, the glycolytic capacity may decrease (Lejeune et al., 2022). The succinate could also be involved in both oxidative phosphorylation and the TCA cycle to produce energy. Moreover, in the anoxic and frozen storage conditions, these two processes might be inhibited to some extent. Oxidation has been reported previously as a mechanism of causing loss of variability in freeze-dried cells by Castro et al. (1997). The absence of oxygen during the freeze-drying process is very important because lipid oxidation might cause more severe membrane permeability and affect the enzymatic activities associated with the membrane (Zheng et al., 2023). Therefore, in this study, considering the microorganisms in the JZH starter, the AAB may play an important role in the process of oxygen consumption for their cell membrane protection. Moreover, it is inferred that the worse cell viability of yeast and LAB in the LMH0 sourdough may be related to the oxidation stress under freezing conditions when the LM starter was stored at −20°C for 6 months, while some of these oxidation stresses were probable relieved by the AAB in the JZH starter.

ABC transporters could promote the transport of amino acids, sugars, and other nutrients into the cell and helped detox the cells by expelling substances that are not conducive to cell growth, such as antibiotics and fatty acids, out of the cell (Qu et al., 2020). The secretion of bacterial toxins by *Lactobacillus* mainly depends on the excretion system of ABC transporters, which help *Lactobacillus* adapt to freeze-drying (Prasad et al., 2016). The enrichment of the membrane of lactobacilli in UFA (unsaturated fatty acid) was positively related to cryotolerance, whereby freeze-resistant bacteria exhibited a higher content of UFAs. Furthermore, the UFA/FA and CFA (cyclic fatty acid)/FA ratio in the membrane are critical factors in cold shock, and LAB transforms a part of UFA into CFA under cold stress (Gao et al., 2021). Conversion of the UFA to their cyclopropane derivatives serves as a protective measure against cold shock (Zhang et al., 2013). In addition, cells containing GSH showed a higher proportion of UFA in cell membranes upon long-term cold treatment (Zhang et al., 2010). The gene abundances of “ABC transporter” and other genes all increased in JZH8, indicating that the vitality of the functional strains may be close to the level before freezing after fermentation. The gene abundance of “ABC transporter” both decreased in JZH0 and LMH0 sourdough samples, so the frozen storage treatment might inhibit the growth of the microbial cells enriched in the genes of ABC transporters. The proportion increased from 0.009% after fermentation in JZH8, but it did not change in the sourdough of LMH8 compared to that in the LMH0 by contrast.

Assimilatory sulfur metabolism is an essential anabolic part of microbial cells, given that the synthesis of oligopeptides (GSH), amino acids (Cys and Met), and co-factors (CoA) all need sulfur elements. Cys is produced as a result of the consequent generation of Met and GSH (Saito, 2004; Wu et al., 2021). After freeze-drying, microbial cells often accumulated intracellular trehalose and retained nearly 100% viability, hence reaching a markedly prolonged shelf life. However, none of these effects were observed with exogenously added trehalose (Termont et al., 2006). In this study, the trehalose contents of the JZH starter reached more than twice of that of the LMH starter samples (2.203 and 1.014 mg/g, respectively). *L. sanfranciscensis* DSM20451 cells containing GSH displayed a higher resistance against cold stress induced by freeze-drying, freeze–thawing, and 4°C cold treatment than those without GSH (Zhang et al., 2010). Therefore, in this study, the abundance of the glutamate–cysteine ligase gene in LMH0 was 52, 544, whereas that in the LM0 sourdough was 10. Meanwhile, for the glutathione synthase gene, the abundances were 6, 602 and 2, 484 in JZH0 and JZ0, respectively. Gene abundances of oxidative phosphorylation (ko00190), nitrogen metabolism (ko00910), and sulfur metabolism (ko00920) were all increased in the JZH0 sourdough. Thus, nitrogen and sulfur metabolism are very important in the anoxic and frozen storage conditions, and the energy production by microorganisms in the JZH starter (AAB dominated) was through oxidative phosphorylation, and it mainly depended on the methane metabolism in the LMH starter (LAB-dominated).

The viability of yeast in the frozen dough has a significant relationship with proofing time, loaf volume, and bread porosity because the decrease in yeast gas production and prolonged fermentation time may exist after being frozen (Tsolmonbaatar et al., 2016; Luo et al., 2018). Loss of baking quality in frozen dough has been ascribed to dough weakening and a reduction in both yeast viability and activity (Steffolani et al., 2012), and the quality of steamed bread fermented by frozen starter also decreased, particularly for the LMH starter. The L^*^, a^*^, and b^*^ values indicate lightness ranging from 0 to 100, hue on a red (+) to green (-), and hue on a yellow (+) to blue (-), respectively (Gül et al., 2022). The L^*^ of LMH steamed bread is larger; however, the difference in b^*^ among the different steamed pieces of bread is not significant. Dough stored at −20°C and frozen at 18–39°C/h resulted in higher specific volume and softer bread, but higher rates of staling, and was related to relatively high yeast activity. Moreover, the crust color was lighter and the texture was more uniform for slower frozen dough stored at −20°C. Meanwhile, frozen dough that was stored longer produced a darker and less uniform colored crust (Yi and Kerr, 2009). The hardness and crumb cell features of the steamed bread prepared by the sourdough reactivated after storage at −20°C, an approach that was made by the unstored sourdough (Lattanzi et al., 2014). The hardness of JZH steamed bread is greater than that of JZ steamed bread, but less than that of LM steamed bread. Hence, the addition of a starter stored under frozen conditions for 6 months like LMH and JZH all decreased the softness of the steamed bread. However, the quality of steamed bread prepared by JZ was relatively well-maintained in the JZH steamed bread after the JZ starter was stored at −20°C, while that of the LMH steamed bread quality was the worst.

## Conclusion

The microbial composition (particularly for *Acetobacter*) and the raw material of maize flour in the JZ starter might enhance the survival of the yeast during the frozen storage at −20°C, and the vitality of yeast was better in JZH0 sourdough than in the LMH0 sourdough. Moreover, the FA of the yeast in the JZH8 sourdough was better than that in the LMH8 sourdough. The microorganisms in the JZH starter might adapt to freezing stress by increasing the gene abundances of “oxidative phosphorylation,” “biosynthesis of glutamate,” and “biosynthesis of unsaturated fatty acid” or the proportions of microorganisms enriched in the above genes based on analysis of the shotgun metagenomic sequencing method. Therefore, the fermentation characteristics of yeast and other functional microorganisms in the JZ starter were easier to refresh after frozen storage than that of the LM starter and retained good yeast cell viability with it, thereby suggesting the better application potential of the JZ sourdough fermentation starter in actual production.

## Data availability statement

The datasets presented in this study can be found in online repositories. The names of the repository/repositories and accession number(s) can be found in the article/Supplementary material.

## Author contributions

HL: Funding acquisition, Supervision, Writing—original draft, Writing—review & editing. YL: Formal analysis, Methodology, Software, Writing—original draft. YZ: Data curation, Investigation, Writing—review & editing. XW: Formal analysis, Methodology, Writing—review & editing. XY: Investigation, Writing—review & editing. JQ: Funding acquisition, Resources, Writing—review & editing.

## Supplementary material

Supplementary Figure 1 Pictures of the steamed breads prepared from JZ, JZH, LM, and LMH sourdoughs.
