# Supplementary figures and images for: Fermentation properties and functional stability of dough starter Jiaozi and Laomian after frozen storage
Source: Front Microbiol. 2024 Apr 12;15:1379484. doi: 10.3389/fmicb.2024.1379484 (PMC11046002; doi:10.3389/fmicb.2024.1379484)

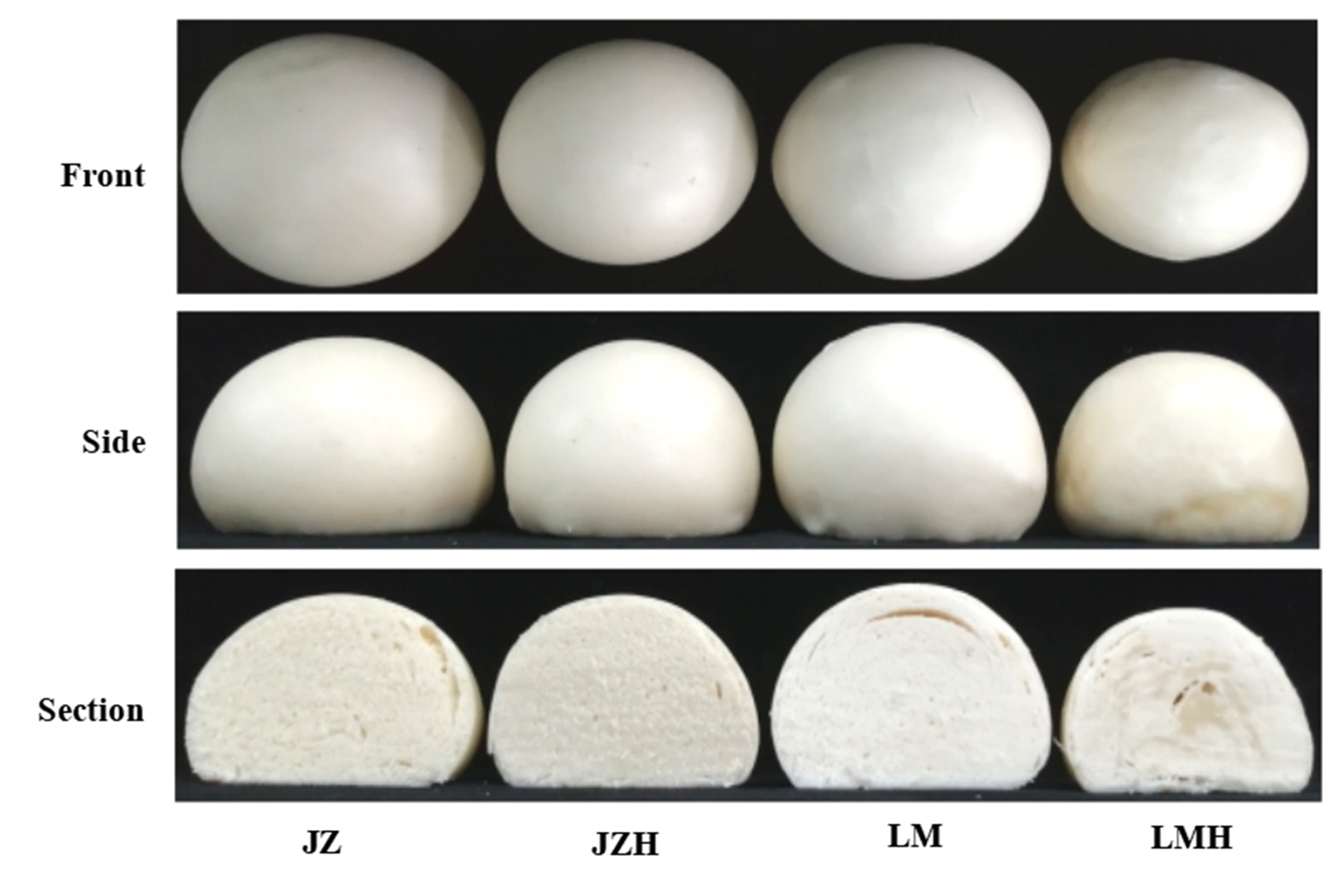

Supplement: Supplementary Figure 1 — Pictures of the steamed breads prepared from JZ, JZH, LM, and LMH sourdoughs. [file Image_1.JPEG]
